# Influence of government-driven quality assessment program on patients with chronic obstructive pulmonary disease

**DOI:** 10.1186/s12931-021-01684-1

**Published:** 2021-03-21

**Authors:** Hye Jung Park, Sung-Ryeol Kim, Sinae Kim, Hye Sun Lee, Bo Yeon Kim, Hye Kyoung Kim, Sang In Ahn, Ji Hyeon Shin, Jae-Hyun Lee, Jung-Won Park

**Affiliations:** 1 Department of Internal Medicine, Gangnam Severance Hospital, Yonsei University College of Medicine, Seoul, Republic of Korea; 2 Division of Allergy and Immunology, Department of Internal Medicine, Yonsei University College of Medicine, Seoul, Republic of Korea; 3 Institute of Allergy, Yonsei University College of Medicine, Seoul, Republic of Korea; 4 Division of Biostatistics, Department of R&D Management, Kangbuk Samsung Hospital, Sungkyunkwan University School of Medicine, Seoul, Republic of Korea; 5 Biostatistics Collaboration Unit, Yonsei University College of Medicine, Seoul, Republic of Korea; 6 Healthcare Insurance Review and Assessment Service, Wonju, Republic of Korea

**Keywords:** Chronic obstructive pulmonary disease, Quality assessment, Prognosis, Clinical outcome, Mortality rate

## Abstract

**Background:**

The Korean Health Insurance Review and Assessment Service (HIRA) has launched the Chronic Obstructive Pulmonary Disease (COPD) Quality Assessment Program (CQAP) since 2014. We aimed to reveal the influence of this national program on clinical outcomes and the burden of COPD in Korea.

**Methods:**

The CQAP is conducted annually. We used healthcare claims data linked with the results of the program provided by HIRA between May 2014 and April 2017. Patients were considered to have COPD if they visited a hospital for COPD management during the assessment term. Those who visited a medical institution for COPD and were prescribed COPD medications at least twice were assessed by the CQAP (assessed subjects, AS; not-assessed subjects, NAS). CQAP evaluated the pulmonary function test conduction rate, regular visitation rate, and prescription rates of COPD medications.

**Results:**

Among the 560,000 patients with COPD, about 140,000 were assessed by the CQAP annually. In both groups, the pulmonary function test conduction rate and inhaled bronchodilator prescription rate improved since 2014. Compared to the NAS group, the risk of admission and all-cause mortality rate in the AS group were significantly reduced by 21.2% and 40.7%, respectively. In patients who were assessed for 3 consecutive years, all of the above variables were high at baseline and were not improved much from implementation of CQAP. In matching analysis, we observed this improvement to be limited in the COPD quality assessment year.

**Conclusions:**

The CQAP by the health insurance bureau has improved the management protocol and prognosis of COPD.

## Introduction

Chronic obstructive pulmonary disease (COPD) is a chronic airway inflammatory disease characterized by a progressive decline in lung function [1, 2]. Therefore, affected patients require close monitoring of symptoms and pulmonary function and engagement in active medical treatment with bronchodilator-based regimen [3]. The worldwide prevalence of COPD is 10.1% with the number of patients continuously increasing, and it also increase the socioeconomic burden [4, 5]. Pulmonary function tests (PFTs) are essential for the diagnosis and follow-up of COPD, and a low FEV_1_ can predict a poor prognosis such as future deterioration. Therefore, periodic PFTs are essential for the optimal monitoring of COPD patients [2].

The Korean Health Insurance Review and Assessment Service (HIRA) has conducted the national Asthma Quality Assessment Program since 2013 to improve the practical management and clinical outcomes of asthmatics [6], and the results of it showed that regular follow-up visits significantly reduced the risk for asthma exacerbation [7]. In 2014, HIRA initiated the National COPD Quality Assessment Program (CQAP) to minimize the national burden of COPD and achieve the optimal treatment strategy to improve the quality of medical services for COPD in Korea. The CQAP assessed several parameters in Korean medical institutions with the following protocol: (a) regular lung function monitoring, (b) regularly visiting, (c) inhaled bronchodilator prescription for COPD patients, (d) admission rate, and (e) emergency department visitation rate. Although the annual CQAP results have been released to the public, the direct and indirect impact of this program on clinical practice has not yet been estimated.

In this study, we investigated the influence of the CQAP on the treatment behavior of clinicians managing COPD, the clinical outcomes of COPD patients, and the national burden of COPD in Korea.

## Methods

### The National COPD Quality Assessment Program

The CQAP was proposed by the Korean government in 2014 and is conducted annually to (a) evaluate how COPD patients are treated in all Korean medical institutions; (b) improve the quality of COPD management protocols in clinics; and (c) improve the clinical outcomes and prognosis of COPD patients. It evaluates the following: (1) PFT conduction at least once during the 1-year assessment term; (2) regular follow-up visitation rate of COPD patients (i.e., at least three visits during the assessment term); and (3) inhaled bronchodilator prescription rate. The inhaled bronchodilator includes LAMA, long-acting β2 agonists (LABA), short-acting β_2_-agonists (SABA), a LABA + LAMA combination, and a LABA + inhaled corticosteroid combination drug.

After excluding institutions with < 10 COPD patients, HIRA rated all medical institutions according to five grades (1–5, with a lower number indicating better COPD management) in accordance with the overall score of the CQAP results (100–80, 1st grade; 80–65, 2nd grade; 50–65, 3rd grade; 50–35, 4th grade; < 35, 5th grade). The overall score was calculated by a weighted average of the PFT conduction rate (40%), proportion of patients with regular follow-up (20%), and inhaled bronchodilator prescription rate (40%).

### Data extraction from claims data linked with the CQAP

Korea has a unique national health insurance system covering all 50 million Korean people. Therefore, records of medical use, prescribed medications, and tests performed are included in the claims data. We extracted data from May 1, 2014 to April 30, 2017.

### Definition of COPD patients from HIRA claims data

COPD patients were defined as those who visited a medical institution (outpatient and/or inpatient department) at least once during the assessment term to manage his/her COPD. COPD patients who met the following criteria during the assessment term were assessed by the CQAP, and they were called as assessed subjects (AS): (1) age ≥ 40 years; (2) ICD-10 codes for COPD (J43, emphysema and J44, other COPD) as the primary diagnosis or first sub-diagnosis; and (3) at least two separate outpatient visits with the prescription of COPD medications or at least one admission for treatment with systemic steroids followed by outpatient visits. In 2014, this case definition of AS (for COPD) was established by the HIRA committee according to the opinions of respiratory and critical care experts in Korea. Since then, this definition has been widely used by many researchers and data analysts [8, 9]. COPD patients who did not meet the above criteria were not assessed by the CQAP, and they were classified as not-assessed subjects (NAS).

### Determination of clinical outcomes

We defined the clinical outcomes as follows: (a) the number of admissions for treatment in a medical institution because of COPD exacerbation (with the ICD-10 codes of COPD [J43 and J44]); and (b) the yearly all-cause mortality rate.

## Study designs

**Fig. 1:**
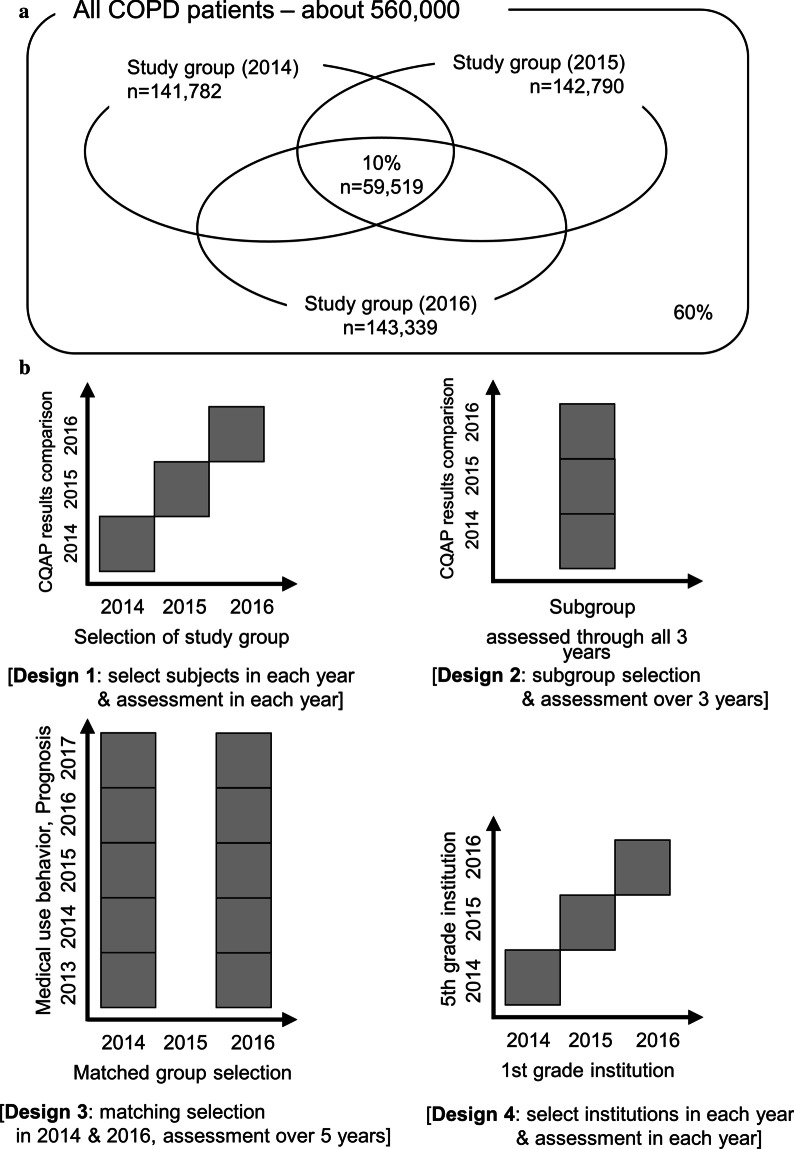
Venn diagram for the COPD population included in this study (**a**) and conceptual diagrams showing four difference study designs (**b**). COPD, chronic obstructive pulmonary disease

Figure 1a briefly depicts the study design and numbers of patients included in this study. We applied four different study models to analyze the CQAP results (Fig. 1b). First, we selected the AS in each year. Next, the NAS groups were created. The CQAP results of the six groups were evaluated and compared at each assessment term. Then, we calculated the risk reduction rate over a 3-year period in the AS group compared with that in the NAS group using the following equation: 1-(relative increase in risk in the AS over 3 years/relative increase in risk in the NAS over 3 years), with a higher positive value indicating positive risk reduction effects of the CQAP in the AS group compared with those in the NAS group. Second, we selected a subgroup of patients assessed by the CQAP for 3 consecutive years and compared their serial annual outcomes. Third, we performed a matching analysis to compare the sequential changes of the CQAP parameters and clinical outcomes of the same patients in 2014 and 2016. To minimize the confounding factors, age, sex, and type of medical institution visited were matched. Fourth, we compared the CQAP results and clinical outcomes of “1st grade” medical institutions (overall CQAP score: 100–80 points) versus “5th grade” institutions (overall CQAP score < 35 points) in the AS and NAS groups, annually from 2014 to 2016. Patients who did not visit any institution or who visited both "1st grade" and "5th grade" institutions were excluded in this design.

### Ethics requirements

The Institutional Review Board of Severance Hospital (Number: 4-2018-0956) approved this study with a waiver for the acquisition of informed consent because of the minimal risk to patients.

### Statistical analyses

We compared demographic data, medical use behaviors, and clinical outcomes between groups using the χ2 test (for categorical variables), independent sample t-test, and analysis of variance test (for continuous variables). To estimate and compare the risk reduction rate over 3 years between the AS and NAS groups, we used a generalized linear model. Propensity score matching was used to reduce the imbalance of age, sex, and types of medical institutions. Data were analyzed using SAS Enterprise version 6.1 (SAS Institute Inc., Cary, NC, USA). A *P-*value < 0.05 was considered to indicate statistical significance.

## Results

### Demographic data, clinical practice parameters, and prognosis of all COPD patients

A total of 569, 253 (2014), 557,682 (2015), and 539,708 (2016) COPD patients were recorded in the HIRA claims data. (Mean age: 67.5–68.2 years, Male: 60%). PFT conduction rates and inhaled LAMA and LABA prescription rates increased steadily each year, while SABA prescription rates steadily decreased for all COPD patients overall (all *P*-values < 0.001). (Table 1).

**Table 1:** Demographics, clinical practice parameters, and prognosis of all COPD patients

Year	2014	2015	2016
COPD patients in HIRA claim data	n = 569,253	n = 557,682	n = 539,708
Demographics			
Age, mean ± SD	66.8 ± 13.2	67.5 ± 13.2	68.2 ± 13.2
Sex, male (%)	349,505 (61.4)	341,160 (61.2)	328,170 (60.8)
Clinical practice parameters			
PFT conduction (%)	191,465 (33.6)	206,519 (37.0)	208,952 (38.7)
Inhaled LAMA prescription (%)	78,044 (13.7)	90,871 (16.3)	94,501 (17.5)
Inhaled LABA prescription (%)	144,130 (25.3)	153,830 (27.6)	148,101 (27.4)
ICS prescription (%)	305,744 (53.7)	295,227 (52.9)	283,804 (52.6)
SABA prescription (%)	90,031 (15.8)	69,349 (12.4)	67,717 (12.6)
Prognosis			
Frequency of admission for COPD /yr, mean ± SD	0.146 ± 0.773	0.147 ± 0.791	0.162 ± 0.869
Medical cost for COPD management, mean ± SD (USD)	335.5 ± 1665.2	375.7 ± 1943.6	423.2 ± 2124.7
All-cause mortality in the next year (%)	17,950 (3.2)	21,114 (3.8)	23,417 (4.3)

SD, standard deviation; PFT, pulmonary function test; LAMA, long-acting muscarine antagonist; LABA, long-acting β_2_ agonist; ICS, inhaled corticosteroid; SABA, short-acting β_2_ agonist

Medical cost was calculated using exchange rate between dollar and Korean won (KRW) in Jan 1st 2020 (1 dollar = 1167.5 KRW)

### Study population

Of the 560,000 COPD patients, approximately 142,000 (25.5%) were annually assessed by the CQAP. Approximately 40% of all COPD patients were evaluated by the CQAP at least once over the 3 years. Moreover, 10% of all patients were assessed for all 3 consecutive years (Fig. 1a).

### Demographic data, clinical practice parameters, and prognosis in the AS and NAS groups

In the AS groups, the PFT conduction rate increased from 60.2% to 69.0%, the inhaled LAMA prescription rate increased from 45.1% to 51.8%, and the inhaled LABA prescription rate increased from 49.4% to 53.4% (all *P* < 0.001). In the NAS groups, the PFT conduction rates were significantly lower than those in the AS groups; however, the rate increased from 24.8% to 27.8% (all *P* < 0.001). Similarly, the inhaled LAMA and LABA prescription rates in the NAS groups were also significantly lower than those in the AS groups (all *P* < 0.001). In contrast, the admission frequencies for COPD and all-cause mortality rates in the AS groups were higher than those in the NAS groups (Table 2).

**Table 2:** Demographics, clinical practice parameters and prognosis of study groups

Year	2014		2015		2016	
Study group (number)	AS-2014 (n = 141,782)	NAS-2014 (n = 427,471)	AS-2015 (n = 142,790)	NAS-2015 (n = 414,892)	AS-2016 (n = 143,339)	NAS-2016 (n = 396,369)
Demographics						
Age, mean ± SD	69.9 ± 10.1	65.8 ± 13.9	70.0 ± 10.1	66.7 ± 14.0	70.1 ± 10.1	67.5 ± 14.1
Sex, male (%)	101,795 (71.8)	247,710 (57.9)	103,882 (72.8)	237,278 (57.2)	106,664 (74.4)	221,506 (55.9)
Clinical practice parameters						
PFT conduction (%)	85,392 (60.2)	106,073 (24.8)	91,084 (63.8)	115,435 (27.8)	98,895 (69.0)	110,957 (27.8)
Inhaled LAMA prescription (%)	63,932 (45.1)	14,112 (3.3)	69,833 (48.9)	21,038 (5.1)	74,254 (51.8)	20,247 (5.1)
Inhaled LABA prescription (%)	70,091 (49.4)	74,039 (17.3)	76,597 (53.6)	77,233 (18.6)	76,508 (53.4)	71,593 (18.1)
ICS prescription (%)	82,331 (58.1)	223,413 (52.3)	81,284 (56.9)	213,943 (51.6)	81,242 (56.7)	202,562 (51.1)
SABA prescription (%)	51,066 (36.0)	38,965 (9.1)	30,158 (21.1)	39,191 (9.5)	29,931 (20.9)	37,786 (9.5)
Prognosis						
Frequency of admission for COPD/yr, mean ± SD	0.307 ± 1.062	0.091 ± 0.634	0.281 ± 1.002	0.100 ± 0.695	0.296 ± 1.065	0.111 ± 0.777
All-cause mortality in the next year (%)	8278 (5.8)	9672 (2.3)	8217 (5.8)	12,897 (3.1)	8341 (5.8)	15,076 (3.8)

AS, assessed subjects; NAS, not-assessed subjects; SD, standard deviation; PFT, pulmonary function test; LAMA, long-acting muscarine antagonist; LABA, long-acting β_2_ agonist; ICS, inhaled corticosteroid; SABA, short-acting β_2_ agonist

### Comparison between the changes in CQAP parameters and risk reduction rate over 3 years

PFT conduction rates and inhaled LAMA and LABA prescription rates increased over the 3 years for both groups. Significant changes in the clinical practice and prescription pattern for most COPD medications (except ICS) were observed in both groups. Furthermore, although the admission frequency because of COPD slightly decreased in the AS group over the 3 years (−3.6%), it increased in the NAS group (+ 22.0%) (risk reduction rate, 21.2%; *P* < 0.001). Finally, the all-cause mortality rate greatly increased in the NAS group over the 3 years (+ 65.2%), whereas it very slightly decreased in the AS group (−0.3%) (risk reduction rate, 40.7%; *P* < 0.001) (Table 3).

**Table 3:** Comparison between the changes in CQAP parameters and risk reduction rate over 3 years

	AS	NAS	Risk reduction rate in AS compared to NAS	*P*-value
Clinical practice parameters				
PFT conduction rate	+ 14.6%	+ 12.1%	− 2.4%	< 0.001
Inhaled LAMA prescription rate	+ 14.9%	+ 54.5%	+ 25.8%	< 0.001
Inhaled LABA prescription rate	+ 8.1%	+ 4.6%	− 3.5%	< 0.001
ICS prescription rate	− 2.4%	− 2.2%	+ 0.2%	< 0.001
SABA prescription rate	− 41.9%	+ 4.4%	+ 44.6%	< 0.001
Prognosis				
Frequency of admission for COPD	− 3.6%	+ 22.0%	+ 21.2%	< 0.001
All-cause mortality in the next year	− 0.3%	+ 65.2%	+ 40.7%	< 0.001

AS, assessed subjects; NAS, not-assessed subjects; PFT, pulmonary function test; LAMA, long-acting muscarine antagonist; LABA, long-acting β_2_ agonist; ICS, inhaled corticosteroid; SABA, short-acting β_2_ agonist

### Serial changes in clinical practice parameters and prognosis in patients assessed by the CQAP for 3 consecutive years

Almost all of the above-mentioned CQAP variables related to clinical practice were high at baseline and remained constant over the assessment period in those evaluated by the CQAP over 3 consecutive years. However, the ICS prescription rate decreased and the admission frequency significantly increased (Table 4).

**Table 4:** Serial changes of clinical practice parameters and prognosis in subjects assessed by CQAP for consecutive 3 years

Year	2014	2015	2016	3-year changes
Overlapping patients in AS group for 3 years	n = 59,519	n = 59,519	n = 59,519	(%)
Clinical practice parameters				
PFT conduction (%)	40,258 (67.6)	39,390 (66.2)	40,831 (68.6)	+ 1.5
Inhaled LAMA prescription (%)	36,700 (61.7)	37,851 (63.6)	36,907 (62.0)	+ 0.5
Inhaled LABA prescription (%)	35,746 (60.1)	37,800 (63.5)	36,656 (61.6)	+ 2.5
ICS prescription (%)	33,468 (56.2)	32,118 (54.0)	32,231 (54.2)	-3.6
SABA prescription (%)	15,029 (25.3)	14,248 (23.9)	14,516 (24.4)	-3.6
Prognosis				
Frequency of admission for COPD/yr, mean ± SD	0.288 ± 0.923	0.277 ± 0.969	0.372 ± 1.260	+ 29.2

AS, assessed subjects; PFT, pulmonary function test; LAMA, long-acting muscarine antagonist; LABA, long-acting β_2_ agonist; ICS, inhaled corticosteroid; SABA, short-acting β_2_ agonist

### Matching analyses

**Fig. 2:**
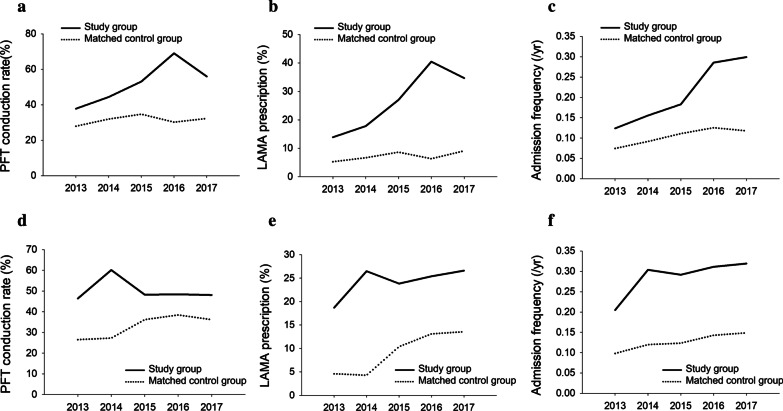
Matching analyses. Comparison of annual changes in the PFT conduction rate (**a**), inhaled LAMA prescription rate (**b**), and admission frequency (**c**) between those patients assessed by CQAP and matched patients that were not assessed in 2016. Comparison of annual changes in the PFT conduction rate (**a**), inhaled LAMA prescription rate (**b**), and admission frequency (**c**) between patients assessed by CQAP and matched patients that were not assessed in 2014. PFT, pulmonary function test; COPD, chronic obstructive pulmonary disease. CQAP, COPD quality assessment program

Matching analyses were conducted in the NAS-2016 and AS-2016 groups (n = 127,927, respectively) and NAS-2014 and AS-2014 groups (n = 137,402, respectively). Although the PFT conduction rate and inhaled LAMA prescription rate in matched AS-2016 patients peaked in 2016, they decreased to the level of them in 2017. However, the admission frequency in the matched AS-2016 patients increased in 2016 and continued increasing in 2017. In contrast, the above values were not greatly influenced by the assessment term in the matched NAS-2016 group (Fig. 2a–c). Similar results were observed in the matching analyses between the NAS-2014 and AS-2014 groups (Figs. 2d–f).

### Comparison of clinical practice parameters with the prognosis of COPD patients according to the grade of the medical institution regularly visited

**Fig. 3:**
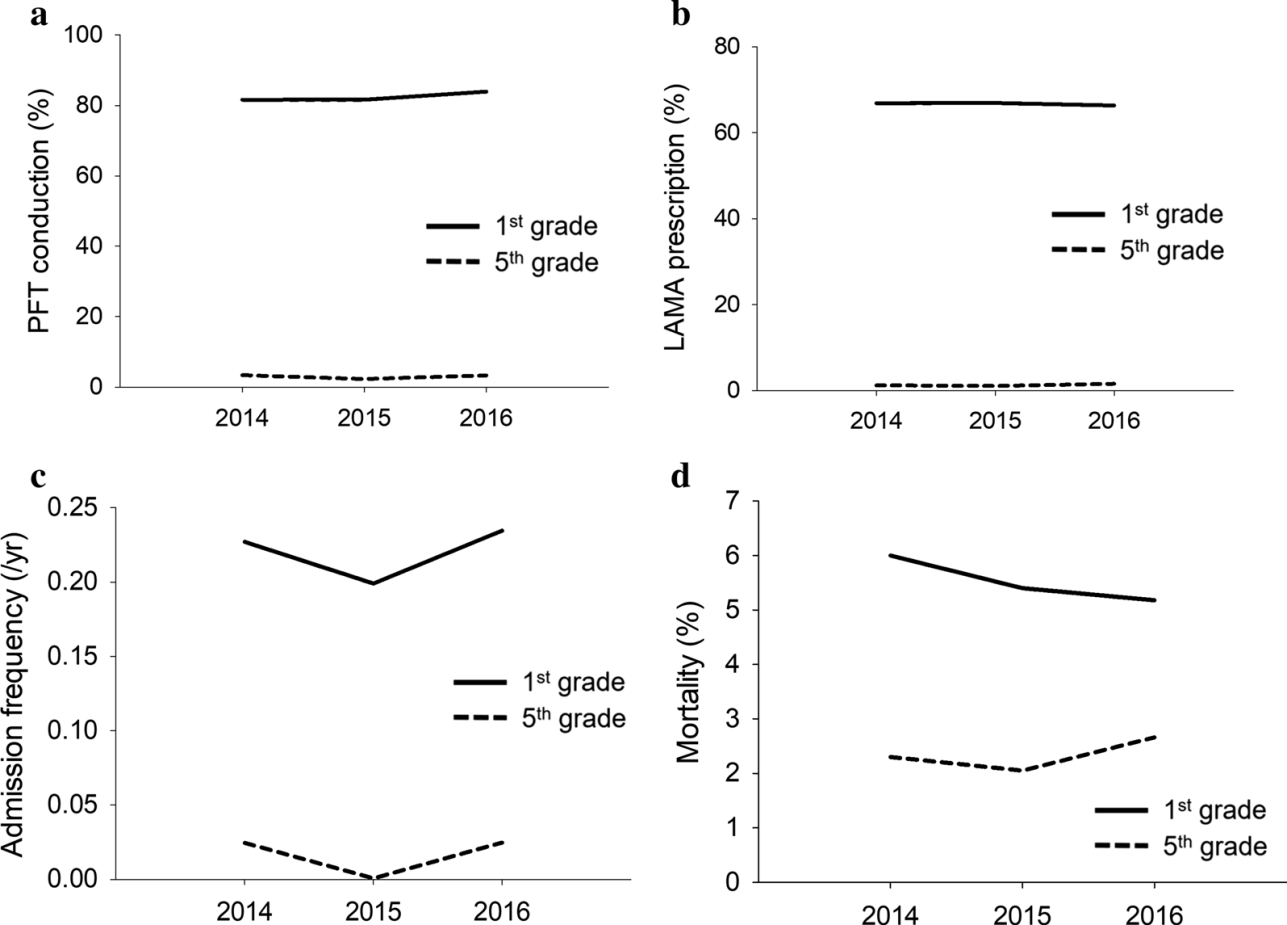
Comparison of annual changes of PFT conduction rate (**a**), inhaled LAMA prescription rate (**b**), and admission frequency (**c**) and all-cause mortality (**d**) between CQAP enrolled patients who visited the “1st grade” medical institution and those who visited the “5th grade” institution. PFT, pulmonary function test; COPD, chronic obstructive pulmonary disease. CQAP, COPD quality assessment program, LAMA, long-acting muscarine antagonists

In the AS groups, COPD patients who visited the “1st grade” medical institutions reported higher PFT conduction rates and an improvement of this rate (81.6% to 84.0%) than those who regularly visited the “5th grade” institutions (3.4% to 3.3%). Rates of inhaled LAMA prescription were also higher among the former patients than among the latter patients. However, the mean admission frequency because of COPD was higher among patients who visited “1st grade” institutions than among the patients who visited “5th grade” institutions (*P* < 0.001). The all-cause mortality rate was higher among the former patients than among the latter patients (*P* < 0.001). The mean risk ratio of mortality to admission frequency for the 3 years was about four times higher among the former patients than among the latter patients (Fig. 3).

## Discussion

The CQAP conducted by HIRA in Korea is a government-driven specific disease-oriented management qualifying program. It aims to assess the level of management protocols achieved for COPD patients in all medical institutions in Korea. HIRA publishes the results of the CQAP annually with information regarding the COPD management quality of each medical institution and ratings from “grade 1” to “grade 5” [10]. In this study, we revealed that the Korean CQAP positively influenced the quality of COPD medical services. Since the start of this program in 2014, the PFT conduction and inhaled bronchodilator prescription rates have steadily increased. Furthermore, these improvements were greater in the AS group than in the NAS group. It can be assumed that the CQAP has some influence on clinicians’ practices and patients’ medical use behavior in real-world settings.

The CQAP also influenced COPD management and prognosis. This phenomenon appeared differently in the AS and NAS groups. Every year, the CQAP data showed that the percentage of prescribed bronchodilators in the AS group was higher than that in the NAS group*.* Every year, the it also showed that the admission rate of patients in the AS group because of COPD was higher than that of patients in the NAS group; the mortality rate was also higher in the AS group. It is thought that patients with more severe COPD were included in the AS group than in the NAS group. We speculate that this phenomenon is because the coverage of medical insurance increased with national income and the interest in the health of chronically ill patients increased. Additionally, because of the natural course of COPD, the mortality rate of severely ill patients is inevitably high.

However, when analyzing the results for 3 years in a row, the hospitalization and mortality rates in the AS group slightly decreased, while in the NAS group both rates increased significantly. The risk reduction rates of the AS group for this result were 21.2% and 40.7%, and thorough COPD management through the CQAP played a major role. The guidelines suggest that the use of inhaled bronchodilators has a positive effect on the management and prognosis of COPD patients, but they are not widely used in practice in Korea. This implies that both clinicians and patients need to change their perceptions about long-term management goals of COPD [11]. Additionally, it is well known that periodic monitoring using PFTs can predict a patient’s prognosis, but it is doubtful whether they are performed in real practice situations [12].

To reduce the socioeconomic burden of chronic illnesses, the physicians managing chronic illnesses should manage patients in the most efficient way possible (with regards to factors such as guidelines and action plans), and patients should show a high degree of adherence to the control and rescue medications [13, 14]. The CQAP is considered a new model for inducing changes in the behavior of doctors and patients. For asthma, HIRA has been conducting the Asthma Quality Assessment Program (AQAP) since 2013. A 4-year AQAP analysis showed that the risk reduction rates of admission frequency because of asthma exacerbation and all-cause mortality rate of the patients observed by AQAP (AS group) were 17.1% and 24.4%. when compared with not assessed subjects (NAS group) [15]. These were lower than those of COPD because the decline in lung function and irreversible changes are more severe in COPD than in asthma, and the probability of systemic co-morbidity is higher in COPD.

However, weak effects were observed in the subgroup assessed by the CQAP for 3 consecutive years. This subgroup had higher PFT conduction and prescription rates of LABA and LABA inhalers at baseline. A higher admission frequency was found compared to those in the NAS group. This subgroup required multiple COPD medications and was highly dependent on high-quality (upper rather than secondary hospital) medical services, implying that this subgroup experienced more severe COPD. Unfortunately, this group did not much improvement.

Additionally, the positive effects of the CQAP may be temporary. In the matching analysis, the PFT conduction and LAMA prescription rates of the AS-2016 group showed a sharp decrease in 2017. Such a phenomenon was also observed in the AS-2014 group. This means that government-led policies alone have limits to the duration of effects on the positive behavior changes of physicians. Therefore, in continuously operating programs such as the CQAP, through the close cooperation with academia as a private sector, doctors themselves can autonomously modify their practices for improvement.

HIRA classify all medical institutions in Korea into five grades considering management protocol of COPD, and made this information publicly available. We can expect 1st grade institutions show better practice performance the compared to the 5th grade institutions, however, the hospitalization and mortality rates increased as the grade of the institution decreased (5th to 1st grade). This can be explained by considering the size of the medical institutions and the severity of COPD. Overall, Korean COPD patients are more likely to be treated by higher-level institutions for more severe illnesses because of continuous pulmonary dysfunction, and are monitored and managed according to global guidelines. Therefore, how CQAP guides mild-to-moderate patients to be treated and monitored according to guidelines at 3rd-, 4th-, and 5th-grade institutions is the main question to be focused in the future.

Although it seems that the prognosis of patients who visit 1st grade institutions is poorer than that of patients who visit 5th grade institutions, we confirmed that improvement of PFT conduction is better in the former. In addition, while admission frequency in the former is about 10 times higher than in the latter, the mortality rate in 1st grade institutions is only about 2.5 times higher. This implies that the mortality risk of patients admitted to 1st grade institutions is only about one quarter of that of those admitted to 5th grade institutions. We found that institutions highly rated by HIRA showed a better prognosis than those rated lower. Therefore, we need to encourage clinicians to improve COPD management protocols as suggested by HIRA to improve their rating because it will help improve the prognosis of COPD patients.

The NAS group was younger and consisted of more females compared to the AS group. The NAS group might include mild COPD patients who did not need COPD medication regularly. Because aging and smoking history are critical factors for development and progression of COPD, young female patients might have relatively mild COPD. Additionally, female COPD patients might be ashamed and afraid to be diagnosed with COPD. Then, they may not visit the hospital or be frequently medicated for COPD. We speculated these reasons might lead to NAS group to be predominantly consisting of young females.

The percentage of prescribed ICS in the AS and NAS groups was similar, whereas percentage of prescribed bronchodilator in the AS group is higher than that in the NAS group. This could mean that the NAS group did not have proper management (including bronchodilator), and they used ICS or oral medication to relieve their symptoms. Additionally, we did not exclude asthma patients to clarify real-world characteristics of COPD. Consequently, these might lead to a high percentage of prescribed ICS.

This study has strengths from several perspectives. First, this is the first study to reveal a positive influence of the national health insurance program and the CQAP, and an improvement in the overall prognosis of COPD using four different study designs. Second, we assessed the complete national insurance cohort HIRA database to reduce selection bias. Third, it reveals the multi-faceted positive influences of the CQAP using a variety of study designs.

This study has some limitations. First, COPD patients were defined with diagnostic codes only. Second, this study was not designed with randomization; thus, the direct effects of the CQAP could not be ascertained. Third, the study results should not be interpreted broadly for application in countries that do not implement a national medical insurance system. Fourth, there is the possibility of an unmeasured negative impact of the CQAP (example: the intentional removal or insertion of diagnostic codes, over-prescription of COPD medications, and avoidance of non-adherent patients). Last, we did not define COPD exacerbation by medication use including antibiotics or steroids.

## Conclusions

We found a positive influence of the CQAP conducted by government on both the patient management protocol and prognosis of COPD in Korea. The CQAP was found to be effective overall, but restricted to the annual assessment term. Thus, we believe that the continued application of this type of quality assessment program will help improve clinical outcomes and medical utilization for COPD patients.

## Data Availability

The datasets used and analysed during the current study are available from the corresponding author on reasonable request.

## Abbreviations

AS: Assessed subjects
CI: Confidence interval
COPD: Chronic obstructive pulmonary disease
CQAP: COPD quality assessment program
GOLD: Global Initiative for Chronic Obstructive Lung Disease
HIRA: Health Insurance Review and Assessment Service
ICS: Inhaled corticosteroids
LABA: Long-acting β_2_ agonists
LAMA: Long-acting muscarine antagonists
NAS: Not-assessed subjects
OR: Odds ratio
PFT: Pulmonary function test
SABA: Short-acting β_2_-agonists
